# A New View of the Nature of Inflammation, with Cases of Croup and Bronchitis, Illustrating a Simple and Successful Mode of Treatment, and of the Use of the Thymus Gland, &c.

**Published:** 1834-10-01

**Authors:** 


*A New View of the Nature of Inflammation, with Cases of Croup and Bronchitis, illustrating a simple and successful Mode of Treatment, and of the Use of the Thymus Gland, &c.* By William Forrester Bow, m.d.—Edinburgh, 1834. 8vo. pp. 94.

Several of the cases given by Dr. Bow, in this little work, appeared a few years since in the pages of the London Medical and Physical Journal: they must have startled some medical theorists, and many of the so-called practical men, or followers of routine. Dr. Bow, dissatisfied with the usual methods of treating croup and bronchitis in children, tried the use of an opiated liniment, instead of leeches or other modes of depletion; and this new plan succeeded so well, that he has continued it to the present time, and it has been employed by several other practitioners with equal advantage. Dr. Bow prefaces the account of his cases with the physiological theory on which his practice is based; but we must hasten on to the purely practical part, as most interesting to the majority of our readers, and refer those who are discontented with our omissions to Dr. Bow's very ingenious speculations, as detailed in the treatise itself. Some of the more vigorous professors of leechcraft will certainly be astounded at the following cases.

"Case i. 14th February, 1831. —— Darling, aged nine months, was reported at noon, by Mr. Fender, to be labouring under bronchitis. I immediately proceeded with him to the patient; found the breathing difficult, the inspirations being short and frequent, accompanied with wheezing; the face very pale, the lips having a purple tinge; skin exceedingly hot; hoarseness of the voice when the child cried; pulse rapid. The child had been ill for some days, but the mother, thinking that nothing ailed it, except a common cold, did not become alarmed until this morning. Nothing had been prescribed before I saw it. Two grains of calomel were given, and the breast and back rubbed with rather more than a drachm of opiate liniment.*

"The calomel was rejected from the stomach almost as soon as taken. After the application of the liniment, the child fell into a sound sleep; at two o'clock he awoke, and sucked greedily; and at our visit, a little after two, we found him in a profuse perspiration, the voice perfectly free from hoarseness, and the breathing comparatively easy. At four o'clock the breathing had again become difficult, but in degree nothing equal to what it had been in the morning. At six o'clock the child seemed calm and con-

"* The liniment I used was as follows; I have since added a little soap: ℞. Opii, ʒj.; Linim. Camph. c. ℥viij. Digere per dies aliquot et effunde linimentum."

tented; the eyes were sprightly, and some colour had returned to the cheeks. A portion of liniment was left with the mother, with orders to apply it, should the breathing again become hurried.

"15th February. About three o'clock this morning the liniment was applied, as the mother thought the state of the breathing required it. At our visit, at ten A.M., we found that the bowels had been twice moved, the effect of the calomel. The child seemed quite well, and therefore nothing was prescribed. At seven P.M. the child continued well. At the request of the mother, some of the liniment was left, to be applied, if necessary.

"16th. The liniment was not applied. Cured." (P. 31.)

"Case v. 24th February, 1833. John Robson, aged ten months, was seen by me this morning at six A.M. He had been colded and fretful for some days, but not until last night did his mother deem him seriously ill. I found the breathing laboured, with wheezing; great heat of skin; pulse rapid; face pale, colour of the lips inclining to purple. He had slept little or none during the night, and was so restless that his mother was forced to pace the room with him in her arms all that time. He frequently took the breast, but it almost always produced coughing.

"I had a vial of liniment in my pocket, and saw applied rather more, I think, than three drachms of it.

"Ten o'clock A.M. About half an hour after the liniment was applied he fell asleep; he was then put to bed, and is still sleeping. Pulse 130; inspirations, as denoted by the movement of the bedclothes, 72; no wheezing. Some colour has returned to the cheek; skin moist.

"Two drachms of liniment to be applied when the child awakens, and two grains of calomel to be given.

"Four o'clock P.M. About an hour after my last visit the liniment was applied, and the calomel given; after which, the child sucked greedily, and, as the mother said, seemed quite well. He again slept from twelve o'clock until three, and is now in his mother's arms, apparently well. Neither pulse nor inspirations can be numbered with certainty.

"Ten o'clock P.M. The bowels have been three times moved since last visit; the skin is cool and moist; respiration perfectly free; has coughed occasionally since last visit.

"A teaspoonful of liniment to be applied to the back.

"25th February. He has slept almost the whole night, and appears quite well.

"Case vi. 29th March, 1833. Ann Short, aged two months. I was summoned to this infant this morning, but, as it was in the country, and as I could not leave the town, I did not see it. From the description of the symptoms, however, I suspected bronchitis, and prescribed two powders, each containing a grain of calomel; one to be given as soon as possible, the other two hours afterwards. A teaspoonful of the liniment to be applied every two hours.

"At ten o'clock P. M. I was again sent for; the child, I was told, was not expected to live. I hastened to the house, and, arriving there at eleven o'clock, found the cause of alarm to be that two or three times she had been seized with violent coughing, during which, as the parents expressed it, she got black in the face, her eyes becoming fixed. The pulse I could not count, it was rapid; the inspirations were irregular, but seemed to average seventy in the minute; the skin was hot; the face pale; lividity around the mouth and eyes; great restlessness. The calomel had acted on the bowels.

"I caused the child to be stripped, and saw applied to her breast, bowels, and back, about half an ounce of the liniment.

"The clergyman, who had been sent for to baptize the child, then performed his office; and, after waiting half an hour, we took our leave, the child being then asleep.

"30th March. The child passed a good night, and is now apparently quite well; nothing prescribed.

"I happened to see this child about a month afterwards, and was astonished at its fatness; it was the fattest child I ever saw at its age, although before the attack she was considered puny." (P. 35.)

The following case is one of several successfully treated on this plan by Mr. Burn, of Belford.

"CASE X. 20th June, 1831. Mary Clarke, aged three years, has been ill twenty-four hours of a supposed cold: the case, however, is decidedly one of croup, characterized by difficult and sonorous breathing, cough, &c. Three drachms of liniment.

"Nine P. M. The child was immediately relieved by the liniment; indeed, as the mother said, before it was all applied. The breathing is again somewhat heavy, and the cough still croupy. Repeat the liniment, with a dose of calomel.

"21st June. Has slept well all night; breathing relieved; cough easier. Repeat the liniment.

"22d. Quite well." (P. 41.)

After giving his cases, our author proceeds to criticise the observations on croup to be met with in other medical writers; for instance:

"'It is true,' says Dr. Hastings, 'that, for the most part, children do not bear the loss of blood well; but in an attack which is menacing life there is no alternative: we must adopt powerful measures, for without them the disease (bronchitis) will almost certainly prove fatal; but such is its dangerous character, that even by them its progress is often not arrested.' Dr. Hastings is not blind to the injurious effects of exhausting remedies, but it would appear that we have no alternative. We must have recourse, he says, to general bloodletting to diminish the excitement of the heart and larger arteries, and to local bloodletting to relieve the weakened and dilated capillaries. Fortunately for the patients whose cases I have detailed, and for many more, I was not reduced to the sad necessity of taking blood to diminish the excitement of the heart and larger arteries, because I conceived such excitement did not exist. Their pathological condition I considered as arising from weakness, a state similar to that which the smaller arteries of an inflamed part acquire. I had not recourse to local bloodletting to relieve the weakened and dilated capillaries, for, as I conceived, they became dilated in consequence of weakness, I considered it better to enable them to relieve themselves." (P. 48.)

The physiology of inflammation is perhaps not yet in a sufficiently advanced state to allow rules of practice to be deduced from it; yet it is satisfactory when they can be made to coincide with ingenious conjectures and probable analogies, and this is certainly the case with our author's mode of treating croup. He supposes that the liniment is not absorbed, but that, by acting on the cutaneous nerves, "it allays the excitement of the sentient system of nerves, to support which excitement the arterial system had been robbed of its contractility." (P. 60.)

We do not advise those practitioners who are content with the orthodox manner of treating bronchitis and croup, and have taken calomel and leeches for better and for worse, to try Dr. Bow's liniment; but those who have the candour to confess the remarkable fatality of these infantile diseases may safely venture to imitate our author's practice, especially as he tells us that the first half hour will show the power of the narcotic liniment. If it should not succeed, other methods may be tried; and, though delays are proverbially dangerous, so short an interval should be called a pause rather than a delay.

Dr. Bow's conjectures on the physiology of the thymus gland are also very ingenious. He supposes that this organ is a reservoir for the nervous energy suddenly required by the respiratory apparatus at the moment of birth; and he also says that it supplies the thoracic duct with a milky fluid like chyle, and thus contributes to the nourishment of the fœtus. This latter conjecture is also to be found in the work of Sir Astley Cooper, who does not appear to have been aware that it had been previously put forth by Dr. Bow.

The supposition that the thymus is a reservoir for nervous energy, is curiously confirmed by the fact that this gland generally increases in size in hybernating animals, during their period of inaction. Dr. Kopp attributes this to the greater compression of the lungs affording more room for the thymus, (*Medico-Chirurg. Review, July* 1834, p. 197;) but the fact squares singularly well with Dr. Bow's theory, as the lungs, after the torpor of hybernation, have to be refitted, as it were, for a new existence.

This little work is very creditable to Dr. Bow's ingenuity as a physiologist, and practical skill as a physician.

